# Correspondence to: performance of the new SmartCardia wireless, wearable oximeter: a comparison with arterial SaO2 in healthy volunteers

**DOI:** 10.1186/s12871-022-01846-8

**Published:** 2022-09-26

**Authors:** Steven J. Barker, William C. Wilson

**Affiliations:** Masimo Corporation, 52 Discovery Way, Irvine, CA 92618 USA

**Keywords:** Pulse oximetry, Hypoxemia, Sensor

## Abstract

In a recent publication in BMC Anesthesiology, Rincon, et al.present accuracy data for three pulse oximeters with sensors located at three different anatomic sites. Their results for the Masimo Radical with fingertip sensor are erroneous, and we present valid data here. Rincon, et al.show a Bias ± Precision of 2.02 ± 4.6, while the correct laboratory values are -0.01 ± 1.16. The most probable reason for these invalid data is that insufficient time was used at each saturation plateau to allow stabilization of SpO_2_ readings on a fingertip sensor. It has been shown in the literature that fingertip sensors require at least a full minute of stable oxygenation conditions before their readings will be the same as earlobe sensors.

## Main text

Rincon, et al., have published an article comparing the accuracies of three different pulse oximeters: SmartCardia model 7L, Nellcor N-600, and Masimo Radical (model not specified) [1]. The SmartCardia device used an upper-arm sensor, Nellcor an earlobe sensor, and Masimo a fingertip sensor. Healthy volunteers were subjected to stepwise hypoxemic plateaus of 30–60 s duration, with arterial oxygen saturation (SaO2) values going down to roughly 70%. Arterial blood samples were analyzed by CO-oximetry at each plateau (Radiometer ABL-90) as the “gold standard” for accuracy assessment.

**Fig. 1:**
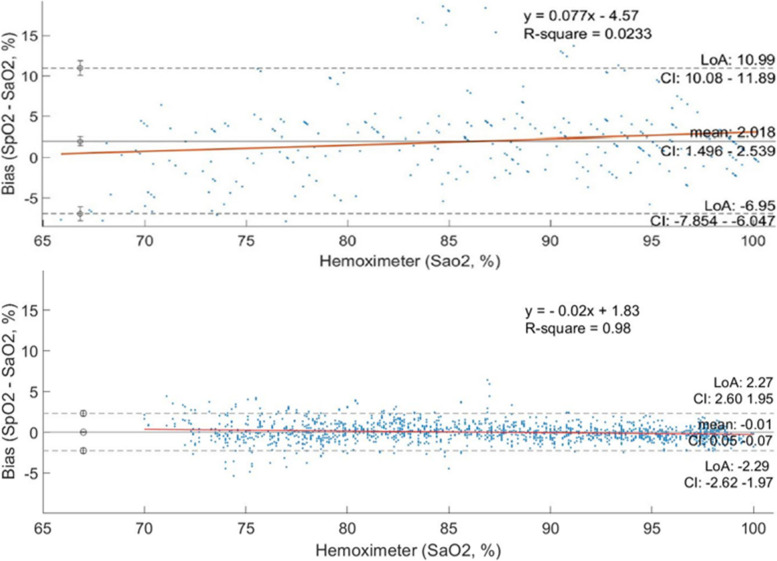
Bias plots showing (SpO2 - SaO2) versus SaO2 for Masimo Radical: Rincon et al.results (top) versus Masimo results (bottom)

The authors show “bias plots” of SpO2 – SaO2 for each of the three pulse oximeters, as well as tabulated results for Bias (mean error), Precision (standard deviation of error) and A_RMS_ (root-mean-square error). The results they show for the Masimo pulse oximeter are totally inconsistent with ClinicalTrials.gov-registered validation data from our laboratories [2]. Rincon quotes a bias ± precision of 2.02 ± 4.6 for Masimo in the full saturation range of 70–100%. The verified and registered values are -0.01 ± 1.16 from a 2017 study. Detailed accuracy statistics comparisons are shown in Table 1. Note that the numbers of volunteer subjects and data pairs are much larger in the Masimo validation dataset. Figure 1 shows the bias plot from Rincon’s paper, compared with the same plot from Masimo laboratories. The difference is obvious.

**Table 1:** Accuracy statistics for Masimo Radical: Rincon et al.results versus Masimo results

	Ricon et al. BMC Anesthesiology (SmartCardia study) 2022 [1]	Masimo (IRB Approved, FDA Cleared Validation Study on health adults) 2017
Bias (Mean Error)	2.02	-0.01
Precisiom (Standard deviation of error)	4.60	1.16
Arms (root-mean–square of the differences)	5.00	1.16
Number of data pairs	286	1493
Number of Subjects	12	25

Close examination of Rincon’s methodology yields a very likely explanation of this discrepancy. They state that their hypoxemic plateaus were maintained “for about 30–60 s at each level.” That is an alarm call to anyone who has performed these desaturation studies. Severinghaus, et al., showed in 1987 that the time delay between oxygenation changes in the lungs and SpO2 readings varies greatly with sensor location, and that delays of a minute or more are common in fingertip sensors [3]. In our own laboratory, we found many subjects with fingertip delays up to two minutes. In contrast, earlobe sensors exhibit a time delay of roughly 10–20 s. The final piece of this puzzle is in Rincon’s Table 1, which suggests that the Masimo fingertip sensor consistently *overestimates* the SaO2. This is logical because their 30–60 s saturation plateaus were being done in a downward sequence; each new plateau had a lower saturation than the previous one. The plateau duration was not sufficient for a finger sensor to reach its stable value before the procedure moved to the next lower plateau.

In summary, the results published by Rincon et al. for a Masimo pulse oximeter with fingertip sensor are incorrect, and a likely cause is the inadequate hypoxemic plateau stabilization time used in this study. We offer no opinions here regarding the accuracies of the results for the other two pulse oximeters in this study.

## Rincon, et al.’s response to the correspondence

Please note that some location sites to measure SpO2 require a longer time period to achieve stabilization plateau than used in this study. For example, fingertip sensors require longer equilibration times to reach steady readings after saturation changes. The literature has shown that fingertip sensors require a full minute or more after an FiO2 change to achieve steady SpO2 values [2]. In this paper, "30 to 60 s" were allowed after each FiO2 change before recording the SpO2 value. This is insufficient time to reach a steady-state reading at some peripheral sites. This paper found a positive bias (tendency to overestimate SpO2) with the Masimo fingertip sensor. This reflects the fact that saturations were varied in descending steps, combined with the insufficient equilibration time.

## Data Availability

The datasets used in this study are available from the corresponding author on reasonable request.

## Abbreviations

SaO2: Arterial oxygen saturation as measured by arterial blood gas analysis
SpO2: Arterial oxygen saturation as measured by pulse oximeter
